# 

**Published:** 1885-09

**Authors:** 


### CONTENTS.

COMMUNICATIONS.

Anæsthetics........ 429
Treatment of Pulpless Teeth........ 433
The Management of Children in the Dental Chair........ 443
The Use of Cocaine in Dentistry........ 448
The Treatment of Deep-seated Abscesses without External Incision 453
Anæsthesia........ 458

PROCEEDINGS.

Report........ 468
SALMAGUNDI........ 472

EDITORIAL.

American Dental Association........ 477
Ohio State Dental Society—Annual Meeting........ 480

